# Composition, Diversity and Sex-Related Differences in Intestinal Microbiota in Captive African Penguins (*Spheniscus demersus*)

**DOI:** 10.3390/ani13132106

**Published:** 2023-06-25

**Authors:** Jingle Jiang

**Affiliations:** Shanghai Endangered Species Conservation and Research Centre, Shanghai Zoo, Shanghai 200335, China; jiangjingle93@163.com

**Keywords:** African penguins, microbiota, sex, 16S rRNA

## Abstract

**Simple Summary:**

The African penguin (*Spheniscus demersus*) is an endangered species. Currently, the intestinal microbiota of African penguins remain uninvestigated. An understanding of the microbial communities in African penguins could provide valuable information for saving this species. Using high-throughput sequencing, we evaluated the composition and diversity of the intestinal microbiota in captive African penguins. Proteobacteria, Actinobacteria and Firmicutes were the predominant bacteria in the intestinal microbiota of captive African penguins. The sex-related differences in microbiota in African penguins were also explored. Female and male African penguins had similar microbial diversities. However, a notable sex-related difference was found between their microbial compositions. Female African penguins have a higher abundance of *Pseudomonas* (a common avian pathogen) than males. Our data suggest that the intestinal microbiota of female African penguins are more unstable than the intestinal microbiota of males in captivity. The intestinal health of female African penguins might require more care in captive management.

**Abstract:**

An understanding of the microbial communities in African penguins (*Spheniscus demersus*) could provide valuable information for saving this endangered species. The objective of this study was to investigate the composition, diversity and sex-related differences in the intestinal microbiota of captive African penguins. Fecal samples were collected from 21 captive adult African penguins reared in the same conditions at Shanghai Zoo. The results show that Proteobacteria, Actinobacteria and Firmicutes were the predominant bacteria in the intestinal microbiota of the captive African penguins. No difference was found in microbial diversity between female and male African penguins, as shown by their similar alpha and beta diversities. However, a notable sex-related difference was found between their microbial compositions. Female African penguins have a higher abundance of *Pseudomonas* and a lower abundance of *Kocuria* than males. A functional prediction indicates that the “mRNA surveillance pathway”, “Polyketide sugar unit biosynthesis”, “Wnt signaling pathway”, “Lysosome” and “Cell cycle” pathways were significantly enriched in the microbiota of female African penguins. In conclusion, the present study indicates that the compositions and predicted functions of the intestinal microbiota are significantly different between the sexes. Our data suggest that the intestinal microbiota of female African penguins are more unstable than the intestinal microbiota of males in captivity.

## 1. Introduction

The intestinal microbiota plays a key role in regulating the host’s intestinal health, the digestion and absorption of nutrients, immunity and other physiological functions [1,2]. The dynamic balance of microbiota is crucial to the survival and environmental adaptation of wild animals [3]. Emerging studies have begun to focus on the composition and diversity of the intestinal microbiota in endangered animals [4,5,6]. Nowadays, research studies on the intestinal microbiota of birds are mainly focused on poultry, and there were limited studies performed on the intestinal microbiota of penguins. The effects of host phylogeny and geographical distribution on the fecal compositions of Adélie penguins (*Pygoscelis adeliae*) were investigated using clone library technology [7]. Studies on the intestinal microbiota of Antarctic penguins have been mainly performed on King (*Aptenodytes patagonicus*), Gentoo (*Pygoscelis papua*), Macaroni (*Eudyptes chrysolophus*) and Little penguins (*Eudyptula minor*) [8]. In addition, the differences in the gastric microbial structures of Chinstrap (*Pygoscelis antarctica*) and Adélie penguins have been analyzed [9]. However, the microbial composition and diversity of African penguins (*Spheniscus demersus*) have not been investigated yet.

African penguins, also known as black-footed penguins or jackass penguins, are mainly distributed on the south and southwest coast of South Africa. Recently, the wild population of African penguins has decreased rapidly due to habitat destruction, the loss of prey populations and environmental changes [10]. The African penguin has been classified as an endangered species by the International Union for Conservation of Nature. Analyzing the composition and diversity of the intestinal microbiota in African penguins is beneficial for understanding the physiological characteristics of this endangered species, and it is extremely important for the protection and management of African penguins.

The influence of the sex of the host on their intestinal microbiota is often ignored [11]. Recently, a number of studies revealed sex-related differences in the structure of intestinal microbiota. Compared with young men, young women have a higher microbial diversity, which may be associated with a higher secretion of sex hormones due to earlier sexual maturity at a young age [12,13]. In animals, the ruminal microbial composition of Tibetan sheep showed notable sex-specific differences, and the relative abundances of Fibrobacteres and Spirochaetes in females were significantly higher than in males [14]. A previous study showed that microbial structure differs markedly between the female and male individuals of three passerine migratory birds (*Tarsiger cyanurus*, *Emberiza elegans* and *Emberiza spodocephala*) [15]. In addition, there is also evidence that significant sex-related differences have been found in the intestinal microbiota of captive Gentoo penguins. The abundance of Firmicutes in the intestines of male Gentoo penguins has been shown to be significantly higher than in females [16]. Herein, we hypothesize that the microbial structure might be discrepant in African penguins of different sexes.

The composition of the intestinal microbiota in animals may be affected by social interaction, diet, living environment and physiological conditions [17]. It is difficult to determine sex-specific microbial differences in wild animals due to numerous interference factors. However, captive animals live in the same controlled environment and eat similar diets in the zoo, which provides suitable conditions for studying sex-related variations in intestinal microbiota in endangered animals.

The purpose of this study was to investigate the composition, diversity and sex-related differences in the intestinal microbiota of captive African penguins using high-throughput sequencing. This research could provide a valuable theoretical basis for evaluating the intestinal health and improving the management of captive African penguins.

## 2. Materials and Methods

### 2.1. Animals and Sample Collection

**Figure 1:**
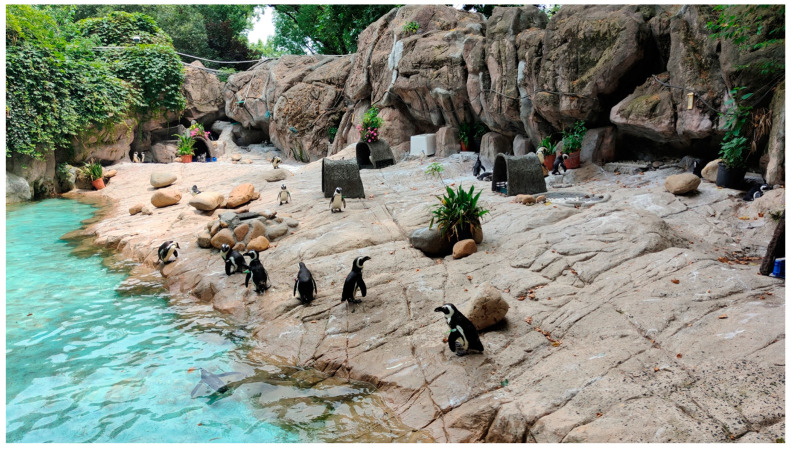
The exhibit of African penguins in Shanghai Zoo.

Fecal samples were collected from captive African penguins reared in Shanghai Zoo (Figure 1). All African penguins used in this study were healthy and lived in the same environment. The age and sex of each penguin are shown in Supplementary Table S1. The sex of each penguin was identified using a feather via polymerase chain reaction (PCR). The routine diet included the little yellow croaker (*Larimichthys polyactis*), capelin (*Mallotus villosus*) and needlefish (*Hyporhamphus sajori*) at a ratio of 5:4:1. The keepers manually evaluated all thawing fishes during their preparation every day to ensure that only fishes of sufficient quality were fed to the penguins. The amount of feed for each animal was about 10% of the African penguin’s body weight per day. The quantity of the captive diet was similar to the average consumption of African penguins in the wild. The diet did not change during the husbandry of all African penguins. All fecal samples from male (the Male group, *n* = 9) and female (the Female group, *n* = 12) penguins were collected on the same day. Previous studies have shown that the composition and diversity of microbiota in birds could alter during breeding or molting seasons [18,19]. Thus, the sampling date was chosen in a non-breeding season, and none of the sampled penguins were molting two weeks before the sampling date. The influences of physiological conditions (breeding or molting) were minimized in the present study. Each fecal sample was collected immediately after excretion and stored at −80 °C. This study was approved by the Ethics and Animal Welfare Committee of Shanghai Zoo (No. 202202).

### 2.2. DNA Extraction and Sequencing of 16S Ribosomal RNA (rRNA) Gene

Total genome DNA was extracted from the fecal samples using a QIAampDNA Stool Mini Kit (Qiagen, Valencia, CA, USA), according to the protocol. The concentration and quality of the total DNA were determined using a ND-2000 micro spectrophotometer (Thermo Scientific, Wilmington, DE, USA). Qualified DNA was diluted to 1 ng/μL using sterile water.

A PCR was performed using Q5^®^ High-Fidelity DNA Polymerase (New England Biolabs, Beijing, China). The 16S V3-V4 region of the 16S rRNA gene was amplified by a specific primer with the barcode (515F, GTGCCAGCMGCCGCGGTAA; 806R, GGACTACHVGGGTWTCTAAT). The PCR products were purified using VAHTSTM DNA Clean Beads (Vazyme, Nanjing, China). The sequencing libraries were generated using a TruSeq Nano DNA LT Library Prep Kit (Illumina, San Diego, CA, USA). Finally, the libraries were sequenced on an Illumina Miseq platform (Illumina, San Diego, CA, USA) with a 300 bp paired-end strategy.

### 2.3. Data Analysis of the 16S rRNA Gene

The reads were qualified, assembled and clustered to the amplicon sequence variants using Vsearch (v2.13.4_linux_x86_64) and Cutadapt (v2.3) software. Sequences with ≥97% similarity were assigned to the same operational taxonomic unit (OTU). Based on the output normalized data of the OTU abundance information, we performed an analysis of alpha and beta diversities. We further performed a linear discriminant analysis effect size (LEfSe) analysis to determine differentially abundant bacteria between the Female and Male groups. Moreover, the 16S rRNA gene sequences were used to calculate and predict the functional pathways in the Kyoto Encyclopedia of Genes and Genomes (KEGG) database via the Phylogenetic Investigation of Communities by Reconstruction of Unobserved States 2 (PICRUSt2) functional prediction.

### 2.4. Statistical Analysis

The indices of alpha diversity (Chao1, Faith_pd, Goods_coverage, Shannon, Simpson, Pielou_e and Observed species) and a rarefaction curve were calculated and created using the Quantitative Insights Into Microbial Ecology 2 (QIIME2) software. A principal co-ordinate analysis (PCoA) was conducted using the QIIME2 software to discriminate between the compositions of the intestinal microbiota of African penguins of different sexes. A hierarchical clustering analysis and PICRUSt2 functional prediction were performed using R language. An LEfSe analysis was performed using R language and Python LEfSe. The Shapiro–Wilk test was conducted to determine the normality distribution of the PICRUSt2 data. A two-tailed Student’s *t*-test was used to compare the results between the Female and Male groups. Differences were considered to be statistically significant at *p* < 0.05.

## 3. Results

### 3.1. Microbial Compositions of African Penguins of Different Sexes

A total of 1,025,348 sequences were produced from 21 fecal samples via Illumina high-throughput sequencing technology. After quality control, denoising and assembly, 853,472 qualified sequences were obtained with an average of length of 410 bp and 40,641 (32,176–55,523) high-quality sequences per sample.

**Figure 2:**
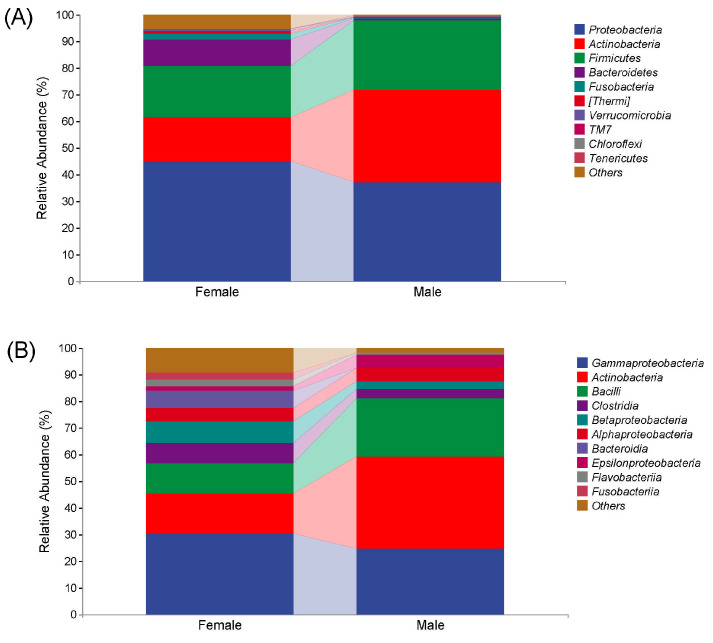
Microbial compositions at phylum (**A**) and class (**B**) levels in captive African penguins of different sexes.

Figure 2 shows the microbial compositions of African penguins of different sexes. In the Female group, at the phylum level, Proteobacteria (45.07%) was the predominant bacteria, followed by Firmicutes (19.37%), Actinobacteria (16.31%), Bacteroidetes (9.80%), Fusobacteria (2.37%), Thermi (1.03%), Verrucomicrobia (0.58%), TM7 (0.08%) and Chloroflexi (0.01%). At the class level, Gammaproteobacteria (30.43%) was the most prevalent bacteria, followed by Actinobacteria (15.15%), Bacilli (11.17%), Betaproteobacteria (7.99%), Clostridia (7.71%), Bacteroidia (6.52%), Alphaproteobacteria (4.99%), Flavobacteriia (2.72%), Fusobacteriia (2.37%) and Epsilonproteobacteria (1.59%).

In the Male group, at the phylum level, Proteobacteria (37.27%) was also the predominant bacteria, followed by Actinobacteria (34.69%), Firmicutes (25.94%), Bacteroidetes (0.81%), TM7 (0.46%), Chloroflexi (0.24%), Thermi (0.10%), Tenericutes (0.08%), Fusobacteria (0.07%) and Verrucomicrobia (0.02%). At the class level, Actinobacteria (34.56%) was the most prevalent bacteria, followed by Gammaproteobacteria (24.69%), Bacilli (21.72%), Epsilonproteobacteria (4.97%), Alphaproteobacteria (4.74%), Clostridia (3.85%), Betaproteobacteria (2.78%), Flavobacteriia (0.73%), Fusobacteriia (0.07%) and Bacteroidia (0.06%).

### 3.2. Alpha Diversity of Intestinal Microbiota in African Penguins of Different Sexes

**Figure 3:**
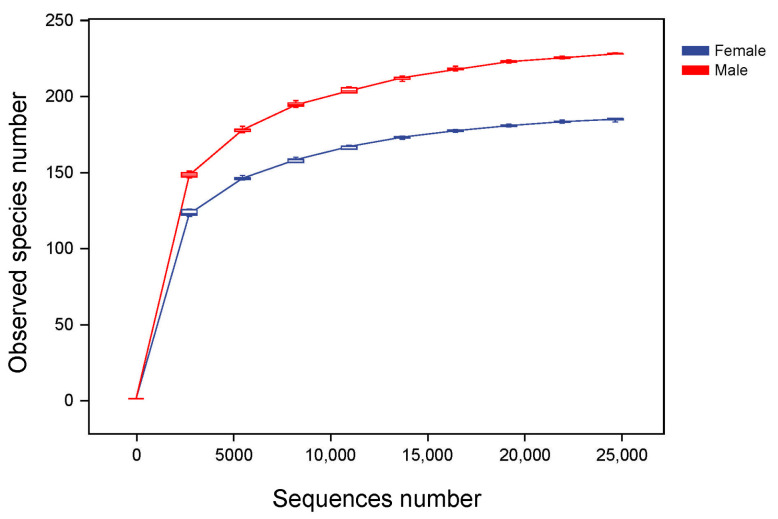
Rarefaction analysis of the microbial community in captive African penguins of different sexes. Reads with 97% similarity are clustered into the observed species number. The sequence number represents the number of sequencing reads.

**Figure 4:**
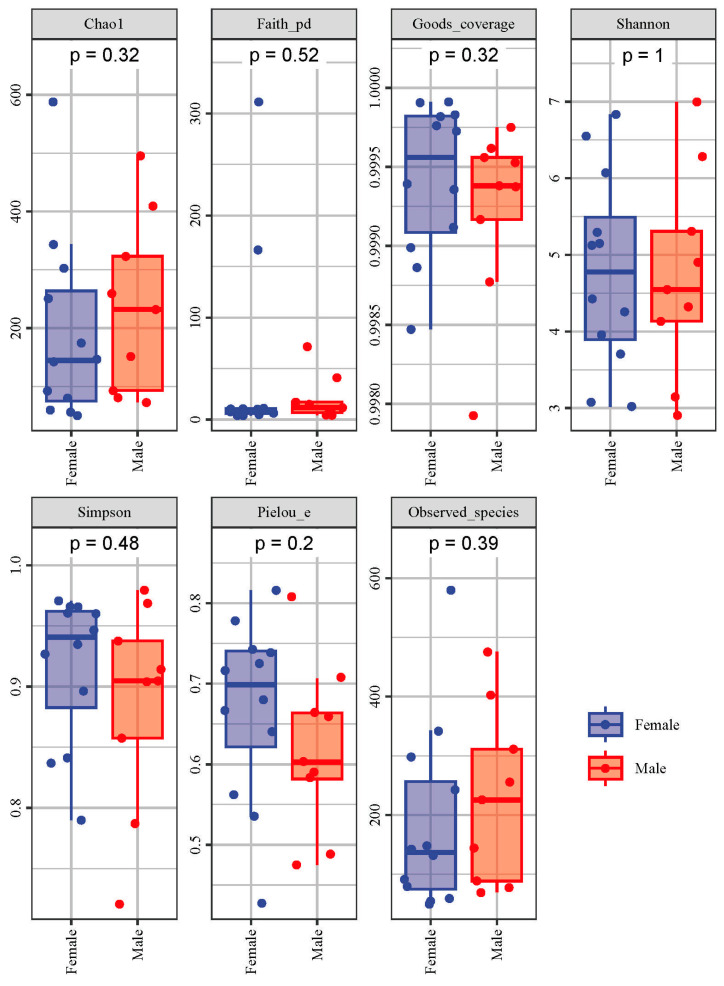
Alpha diversity (Chao1, Faith_pd, Goods_coverage, Shannon, Simpson, Pielou_e and Observed species) indices of intestinal microbiota between female and male African penguins.

Alpha diversity reflects the richness, diversity and evenness of microbiota in a host. Based on the data of the Observed species, a rarefaction curve was created (Figure 3). The rarefaction curves were found to reach the saturation plateau, indicating that the sequencing depth was large enough to estimate the diversity of intestinal microbiota in the African penguins. The indices of alpha diversity are shown in Figure 4. The Chao1 and Observed species indices can reflect the richness of the microbial structure. The Shannon and Simpson indices can evaluate the diversity of intestinal microbiota. The Faith_pd index can measure the evolutionary diversity of the microbial community. The Pielou_e index can represent the evenness of intestinal microbiota. The Goods_coverage index can assess the coverage of sequencing data. In the present study, the Goods_coverage index of each sample was above 0.99, indicating that the sequencing data for all samples are reliable. All alpha diversity indices showed no significant differences between the Female and Male groups, suggesting that the sex factor had no direct influence on the diversity of intestinal microbiota in African penguins.

### 3.3. Beta Diversity of Intestinal Microbiota in African Penguins of Different Sexes

**Figure 5:**
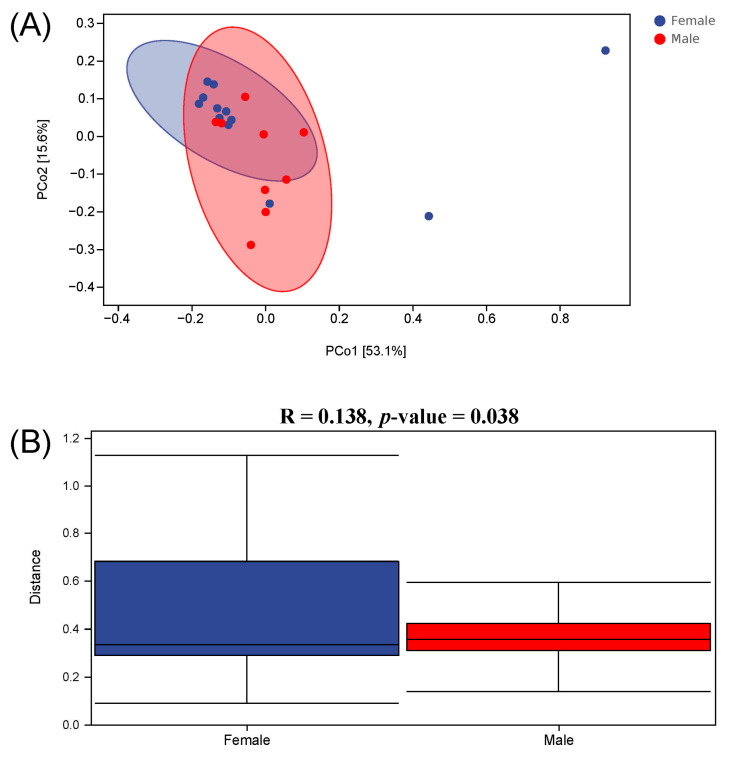
Principal coordinate analysis plots (**A**) and analysis of similarities (**B**) of intestinal microbiota in captive African penguins of different sexes.

**Figure 6:**
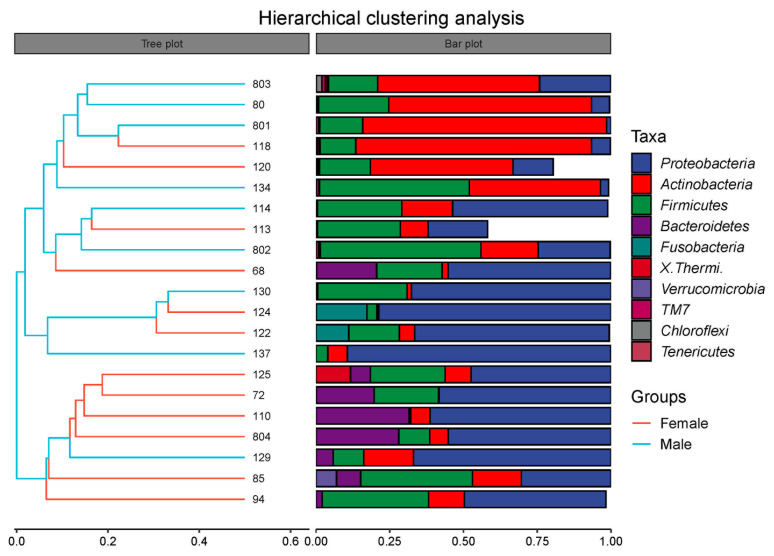
Hierarchical clustering analysis of intestinal microbiota at phylum level in captive African penguins of different sexes.

Beta diversity can reflect the similarity of microbial species in different groups. The result of the PCoA (Figure 5A) revealed that the intestinal microbiota in the Female group was not distinctly separated from the Male group. An analysis of similarities (Anosim) was conducted in order to further elucidate the inter- and intra-group differences (Figure 5B). The result of the Anosim showed that R = 0.138 (*p* < 0.05), suggesting that the difference in beta diversity between the Female and Male groups was significantly greater than the difference within the groups. Moreover, the hierarchical clustering analysis manifested that the intestinal microbiota did not cluster according to the sex factor (Figure 6). This result also demonstrates that female and male African penguins have similar microbial diversities.

### 3.4. Abundances of Intestinal Microbiota in African Penguins of Different Sexes

**Figure 7:**
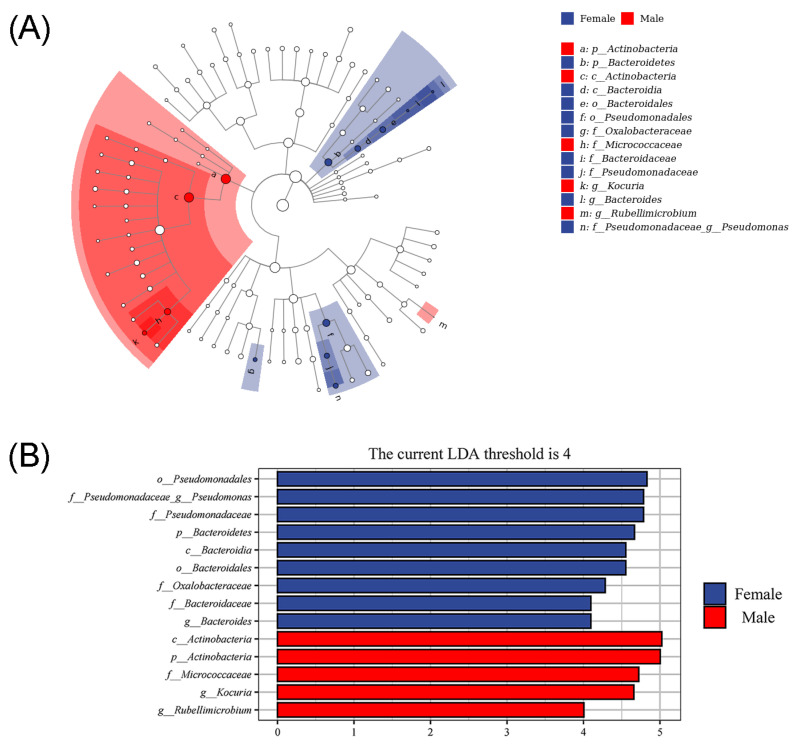
Different structures of intestinal microbiota in female and male African penguins according to LEfSe analysis. (**A**) Cladogram plot of the biomarkers between female and male African penguins. (**B**) Comparison between female and male African penguins. The linear discriminant analysis threshold is 4.

The LEfSe analysis can combine the non-parametric factorial Kruskal–Wallis sum-rank test, Wilcoxon tests and effect size. It is useful for analyzing the differences in all bacteria and discovering markedly altered microbial biomarkers between different groups [20]. Significant taxonomic differences in bacteria (the linear discriminant analysis threshold is 4) are shown in the evolutionary diagram of the LEfSe analysis (Figure 7), suggesting there are sex-related differences in the relative abundances of intestinal microbiota in African penguins. Compared with the Male group, female African penguins had higher abundances of Pseudomonadales, *Pseudomonas*, Pseudomonadaceae, Bacteroidetes, Bacteroidales, Bacteroidia, Oxalobacteraceae, Bacteroidaceae and Bacteroides. Nevertheless, the Female group exhibited lower abundances of Actinobacteria, Actinobacteria, Micrococcaceae, *Kocuria* and *Rubellimicrobium*.

### 3.5. Functional Predictions of Intestinal Microbiota in African Penguins of Different Sexes

**Figure 8:**
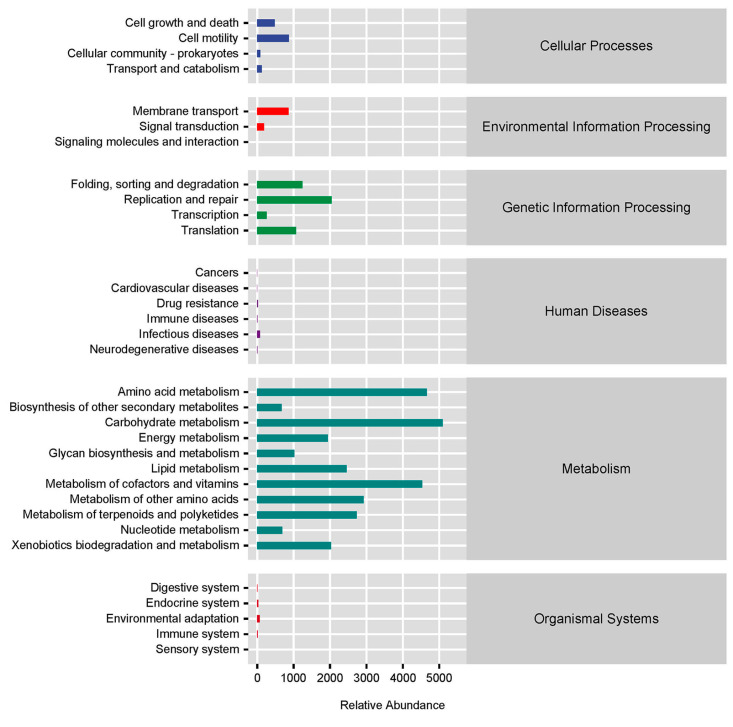
Predicted functional distribution of the intestinal microbiota in captive African penguins of different sexes.

Using the KEGG database, the PICRUSt2 software was applied to predict the microbial function of African penguins. The main functions of intestinal microbiota in African penguins were “Metabolism” and “Genetic Information Processing”. High relative abundances (over 1000) of functional pathways were shown in Figure 8, including “Carbohydrate metabolism”, “Amino acid metabolism”, “Metabolism of cofactors and vitamins”, “Metabolism of other amino acids”, “Metabolism of terpenoids and polyketides”, “Lipid metabolism”, “Replication and repair”, “Xenobiotics biodegradation and metabolism”, “Energy metabolism”, “Folding, sorting and degradation”, “Translation” and “Glycan biosynthesis and metabolism”. A further analysis was conducted to evaluate the predicted functional alterations in the intestinal microbiota in African penguins of different sexes. The functional pathways of “mRNA surveillance pathway”, “Polyketide sugar unit biosynthesis”, “Wnt signaling pathway”, “Lysosome” and “Cell cycle” were significantly enriched in the Female group compared with the Male group (*p* < 0.05, Table 1).

## 4. Discussion

The present study evaluated the composition and diversity of intestinal microbiota in captive African penguins. The results show that the predominant bacteria in African penguins regardless of sex are Proteobacteria (41%), Actinobacteria (25.5%) and Firmicutes (23%). Research studies relating to the intestinal microbiota of penguins have been conducted on wild Antarctic penguins; the predominant bacteria in Chinstrap penguins are Firmicutes (60%), Bacteroidetes (17.5%), Proteobacteria (11%) and Fusobacteria (9%); the predominant bacteria in Gentoo penguins are Fusobacteria (55%), Proteobacteria (18%), Firmicutes (18%) and Bacteroidetes (7%); the predominant bacteria in Adélie penguins are Firmicutes (39%), Actinobacteria (30%) and Bacteroidetes (10%) [21]. Currently, to our knowledge, there has been only one study investigating the intestinal microbiota of *Spheniscus* species (*Spheniscus humboldti*). However, this study did not show the composition of the intestinal microbiota at the phylum level, and its sample size was low (*n* = 4) [22]. A previous study showed that the predominant bacteria in captive Chinstrap penguins are Proteobacteria, Firmicutes, Fusobacteria and Bacteroidetes [23]. The microbial compositions of captive Chinstrap penguins are different from the data from wild Chinstrap penguins, which might be a result of different kinds of food [23]. Since the food and living environments vary between captive and wild conditions, whether the intestinal microbiota of captive African penguins could be recognized as a reference for a wild population of this species requires further investigation.

Compared with Antarctic penguins, the composition of theintestinal microbiota in African penguins is unique. The relative abundances of Proteobacteria and Actinobacteria are quite high. Proteobacteria represents the largest phylum of bacteria and the most unstable bacteria, which are highly sensitive to food or other environmental factors [24]. In mammals, an increase in intestinal Proteobacteria abundance represents an unstable host microbial structure; this phenomenon can be often found in disease states (metabolic disorders and intestinal inflammation) [25,26]. Nevertheless, Proteobacteria is the predominant bacteria in adult African penguins. A previous study also showed that the intestinal Proteobacteria abundance of captive Chinstrap penguins is higher than that of wild Chinstrap penguins [23]. It remains unclear whether this is caused by the captive conditions (the ingestion of thawed fish). Studies on different kinds of captive birds have demonstrated that Proteobacteria are the predominant bacteria in the intestines of chickens (*Gallus gallus*), relict gulls (*Larus relictus*), muscovy ducks (*Cairina moschata*), ruddy shelducks (*Tadorna ferruginea*), demoiselle cranes (*Anthropoides virgo*), whooper swans (*Cygnus cygnus*) and black swans (*Cygnus atratus*) [27,28]. In addition, the relative abundance of intestinal Proteobacteria in captive parrots (mealy parrots, *Amazona farinose*; blue-and-yellow Macaws, *Ara ararauna*; and red-and-green macaws, *Ara chloropterus*) has been suggested to be lower than that of wild parrots (5.5% vs. 22.9%) [29]. Whether Proteobacteria have pathogenicity to penguins requires future elucidation. Actinobacteria belong to Gram-positive bacteria and play a pivotal role in the maintenance of intestinal homeostasis. Numerous members of the Actinobacteria phylum have been indicated as probiotics under pathological conditions; they can protect the intestinal barrier and improve intestinal immune function [30]. Our result of appropriately 30% Actinobacteria in the microbial community of African penguins agrees with a previous finding that Adélie penguins have a 30% abundance of intestinal Actinobacteria [21]. Further studies are required to clarify the correlation between Actinobacteria and intestinal health in penguins. Studies on the intestinal microbiota (the abundance of specific bacteria) of African penguins with different age and health conditions have the potential to explain the interaction between microbial community and intestinal health.

Previous studies on sex-related differences in penguins have been mainly conducted on phenotypic changes, including the diet composition and growth rate of Adélie penguins and the foraging strategy of king penguins [31,32]. There are limited studies investigating the sex-related differences in intestinal microbiota in penguins. A previous study showed that female Gentoo penguins have a higher degree of individual differences in their gut microbiota compared with males [16]. A greater impact of sexual maturity on gut microbiota has also been observed in female Gentoo penguins [16]. The sex-related differences are not only found in the phenotypes and behaviors of penguins but are also exhibited in penguin genetics and microorganisms. The microbial communities of avian embryos are partly inherited from parental birds. During the formation of eggs, the microorganisms in an avian oviduct could transfer into the albumen and colonize in the egg [33,34]. All captive African penguins involved in the present study were born in captivity and fed the same diet. Studies on the intestinal microbiota of captive animals living in the same environmental conditions could eliminate the influences of environment and diet, which is conducive to investigating sex-related microbial differences due to genetic factors.

Our results show that microbial diversity is similar between female and male African penguins, but the LEfSe analysis suggests that the relative abundances of certain bacteria exhibit sex-related alterations. The Female group has a higher relative abundance of *Pseudomonas* than the Male group. *Pseudomonas* has strong abilities of oxidation and drug resistance, and it is widely considered as an opportunistic bacterial pathogen. *Pseudomonas* could cause local infections and even septicemia in an immunocompromised host [35]. As in mammals, *Pseudomonas* is a common avian pathogen. *Pseudomonas* infection could induce a secondary infection due to a reduction in the normal microbial community, immunosuppression, systemic diseases or mucosal damage [36]. In saker falcons (*Falco cherrug*) and psittacine birds, *Pseudomonas* infection can lead to respiratory infection, enteritis, peritonitis and bumblefoot [37]. *Pseudomonas* infection also has a positive correlation with rising mortality in young chickens [38]. In addition, penguins could also be infected with *Pseudomonas*. Serositis, pericarditis, airsacculitis, fibrinous pneumonia, enlarged livers and bumblefoot have been found to be associated with *Pseudomonas* infection in African penguins, Humboldt penguins and Magellanic penguins (*Spheniscus magellanicus*) [36]. Furthermore, the abundance of intestinal *Kocuria* in the female African penguins is significantly lower than in the males. It has been indicated that the abundance of intestinal *Kocuria* could gradually decrease along with an elevated degree of necrotic enteritis in chickens [39]. Our data show that female captive African penguins have higher *Pseudomonas* and lower *Kocuria* abundances than males. It is worthwhile to investigate whether the intestines of female African penguins are more vulnerable than those of males in captivity.

Steroid hormones could affect the composition and diversity of intestinal microbiota. It has been shown that steroid hormones could induce membrane stress responses and influence the virulence of *Pseudomonas* [40]. Testosterone could reduce the diffusivity of *Pseudomonas* in vitro [40]. A number of studies have also demonstrated that Gram-negative pathogens exhibit sex-specific differences in the host [41,42]. A previous study showed that the abundance of intestinal Actinobacteria in men is higher than in women [43]. In accordance with this study, we also found an increased abundance of Actinobacteria in the intestines of male African penguins. Meanwhile, the metabolism of steroid hormones is related to the abundance of the intestinal microbial community. Microbiota can synthesize enzymes involved in the metabolism of steroid hormones, and changes in steroid hormone levels can also promote the growth of certain bacteria [44]. Altered steroid hormone levels in African penguins with different sexes might contribute to the sex-related changes of *Pseudomonas* and *Kocuria* abundance. The application of multi-omics combining serum steroid hormone levels, the intestinal microbiome and the blood metabolome will help ascertain the potential molecular mechanism of sex-related differences in the intestinal microbiota of African penguins.

To highlight the importance of microbial alteration, a PICRUSt2 analysis was performed to predict the function and sex-related functional changes in intestinal microbiota. Consistent with the results of a previous study on Gentoo penguins [16], the main functions of intestinal microbiota in African penguins are the metabolism of various nutrients, including amino acids, energy, lipids and vitamins. It is worth noting that the function of the “Wnt signaling pathway” was significantly enriched in the Female group compared with the Male group. Wnt signaling has a key role in the regulation of cell proliferation and tissue homeostasis. The activation of Wnt signaling could be the original cause of many diseases when the tissues suffer stress or dysfunction [45]. It has been suggested that transient activation of Wnt signaling could promote the death of intestinal stem cells and eventually lead to cancers [46]. Our microbial composition and functional prediction results suggest that the intestinal microbiota of captive female African penguins are more unstable. The intestinal health of female African penguins might require more care in captive management.

In captivity, many factors (increased human contact, reduced space and veterinary medical interventions) might result in distinct microbial species colonizing the intestines of captive animals [47]. Variations in dietary composition and quantity might also play a key role. Due to seasonal alterations in the quantities of available prey, wild African penguins show a marked seasonality in their diet [48]. In addition, wild African penguins have erratic diets owing to the fluctuations in their environment or habitat conditions. Thus, future studies are recommended to investigate the seasonal changes in intestinal microbiota in wild African penguins and the microbial distinction between wild and captive populations.

It has been reported that *Spheniscus* species (African and Magellanic penguins) show female-biased mortality in the wild [49,50]. The exact reasons remain unclear. Herein, the present results suggest that the unstable intestinal microbiota of female African penguins might be a potential cause for high female-biased mortality in the wild. Therefore, it is of great significance to explore a practical solution to properly regulating the intestinal microbiota of captive African penguins. Additionally, functional predictions based on the sequencing of the 16S rRNA gene still could not fully elucidate the functions of different microbial communities [51]. The metagenome is recommended to better analyze the microbial function of African penguins.

## 5. Conclusions

In conclusion, the present study investigated the composition and diversity of the intestinal microbiota in captive African penguins and explored the sex-related differences in microbiota. Proteobacteria, Actinobacteria and Firmicutes are the predominant bacteria in the intestinal microbiota of captive African penguins. Microbial diversity is not altered, while the composition and function of intestinal microbiota are significantly different between the sexes. Female African penguins have higher *Pseudomonas* and lower *Kocuria* abundances than the males. In addition, the “mRNA surveillance pathway”, “Polyketide sugar unit biosynthesis”, “Wnt signaling pathway”, “Lysosome” and “Cell cycle” pathways are significantly enriched in the intestinal microbiota of female African penguins. Our data suggest that the intestinal microbiota of female African penguins are more unstable than the intestinal microbiota of males in captivity.

## Figures and Tables

**Table 1:** Significantly altered functions in the microbial communities in female African penguins (using the Male group as the control group).

Pathway	Description	logFC	*p*-Values
ko03015	mRNA surveillance pathway	3.02	0.024
ko00523	Polyketide sugar unit biosynthesis	2.36	0.004
ko04310	Wnt signaling pathway	2.05	0.004
ko04142	Lysosome	0.67	0.013
ko04110	Cell cycle	0.28	0.009

## Data Availability

Data is contained within the article or Supplementary Material.

## Supplementary Materials

The following supporting information can be downloaded at: https://www.mdpi.com/article/10.3390/ani13132106/s1, Table S1: Information of captive African penguins involved in this study.
