# Supplementary material for: Composition, Diversity and Sex-Related Differences in Intestinal Microbiota in Captive African Penguins (Spheniscus demersus)
Source: Animals (Basel). 2023 Jun 25;13(13):2106. doi: 10.3390/ani13132106 (PMC10339943; doi:10.3390/ani13132106)
Supplement: Supplementary file 1 [file animals-13-02106-s001.zip › animals-2461509-supplementary.pdf]

Supplementary Table S1. Information of captive African penguins involved in this study

Number	Sex	Age
68#	Female	14
72#	Female	14
85#	Female	12
94#	Female	11
110#	Female	11
113#	Female	11
118#	Female	11
120#	Female	10
122#	Female	10
124#	Female	10
125#	Female	9
804#	Female	12
80#	Male	13
114#	Male	11
129#	Male	10
130#	Male	9
134#	Male	8
137#	Male	8
801#	Male	10
802#	Male	9
803#	Male	12
